# Supplementary material for: Application of Airy beam light sheet microscopy to examine early neurodevelopmental structures in 3D hiPSC-derived human cortical spheroids
Source: Mol Autism. 2021 Jan 22;12:4. doi: 10.1186/s13229-021-00413-1 (PMC7821651; doi:10.1186/s13229-021-00413-1)
Supplement: Supplementary file 1 — Additional file 1. Additional Methods, Figures and Tables. [file 13229_2021_413_MOESM1_ESM.pdf]

Application of Airy beam Light sheet microscopy to examine early neurodevelopment in 3D hiPSC-derived human cortical spheroids

Dwaipayan Adhya[1,2]*, George Chennell[1]*, James Crowe[1,3]* Eva P. Valencia-Alarcón[1,3], James Seyforth[4], Neveen A. Hosny[4], Marina V. Yasvoina[5], Robert Forster[4], Simon Baron-Cohen[2], Anthony C. Vernon[1,3], Deepak. P. Srivastava[1,3]§

Supplemental Material:

Supplemental Figures

Supplemental Movie Legend

Supplemental Tables

Supplemental Figures

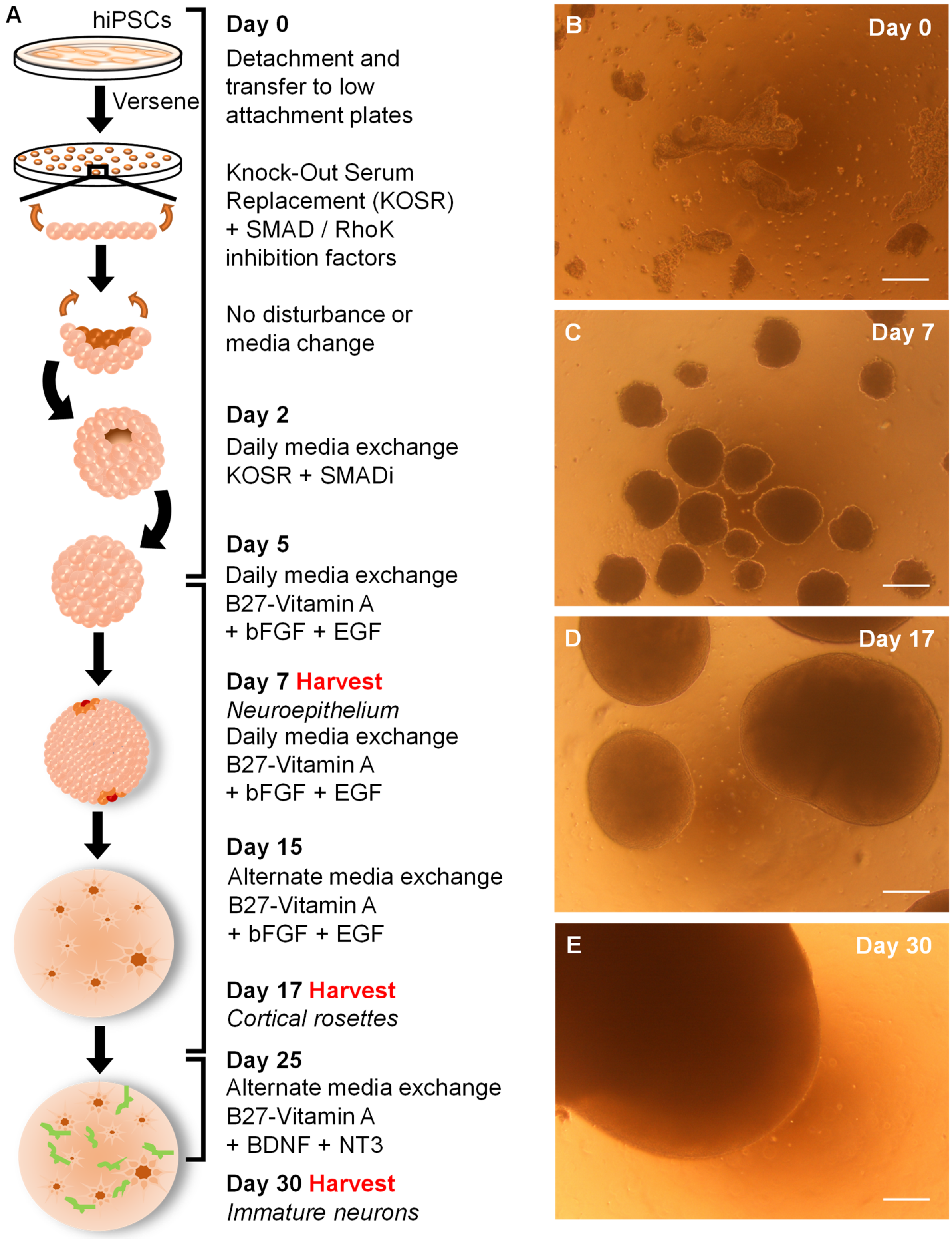

Supplemental Figure 1. Overview of 3D cortical spheroid (hCS) generation. (A) Schematic describing methodology used for 3D neuralization of hiPSCs (adapted from

Paşca et al., 2015). **(B-E)** Bright field micrographs of hiPSCs undergoing 3D neuralization. **(B)** Day 0, confluent hiPSC colonies are detached as intact sheets and transferred to suspension culture to allow 3D morphogenesis alongside dual SMAD inhibition. **(C)** Day 7, an early neuroepithelial fate is achieved and roughly spherical structures form. HCS at day were ~100-300 μm in diameter. **(D)** Day 17, rapid progenitor expansion results in large neuroepithelia formation, with smooth radial organisation at edges as expected during corticogenesis, indicated by white arrows. At this time point, hCS were ~1 mm in diameter. **(E)** Day 30, cortical spheroids approach maximum size, often >2 mm in diameter, with growth factors supporting differentiation to post-mitotic immature neurons. Scale bar = 250 μm.

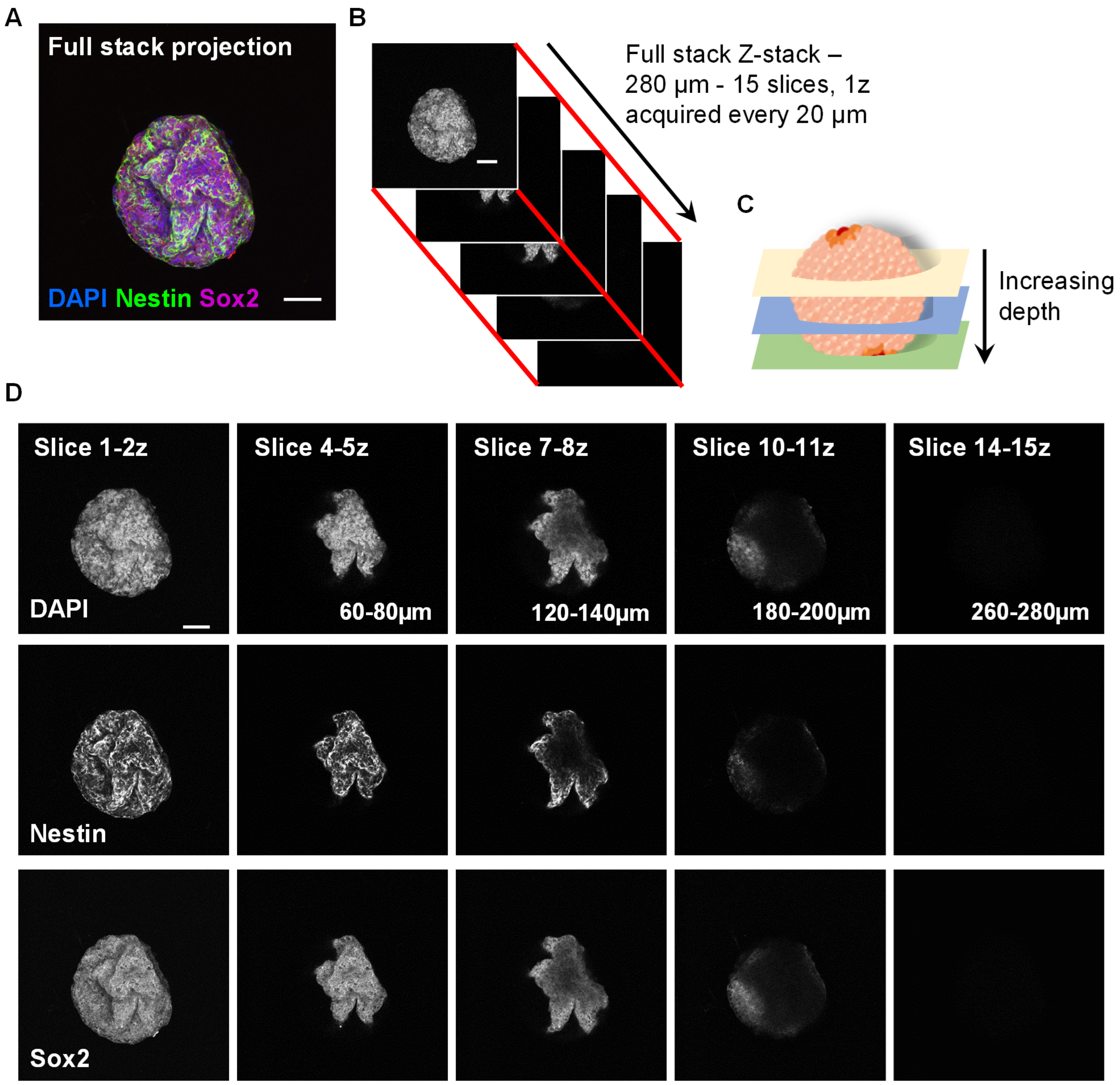

Supplemental Figure 2. Confocal images of day 7 hCS at increasing depths. (A) Composite maximal intensity projection of day 7 hCS shown in Figure 2. **(B)** Visual representation of confocal images that comprise z-stack. **(C)** Schematic of how images within z-stack correspond to overall hCS structure. **(D)** Confocal images of each imaged channel taken at differing depths (axial plane). A significant drop off in intensity is seen after 100 µm. Scale bar = 50 µm.

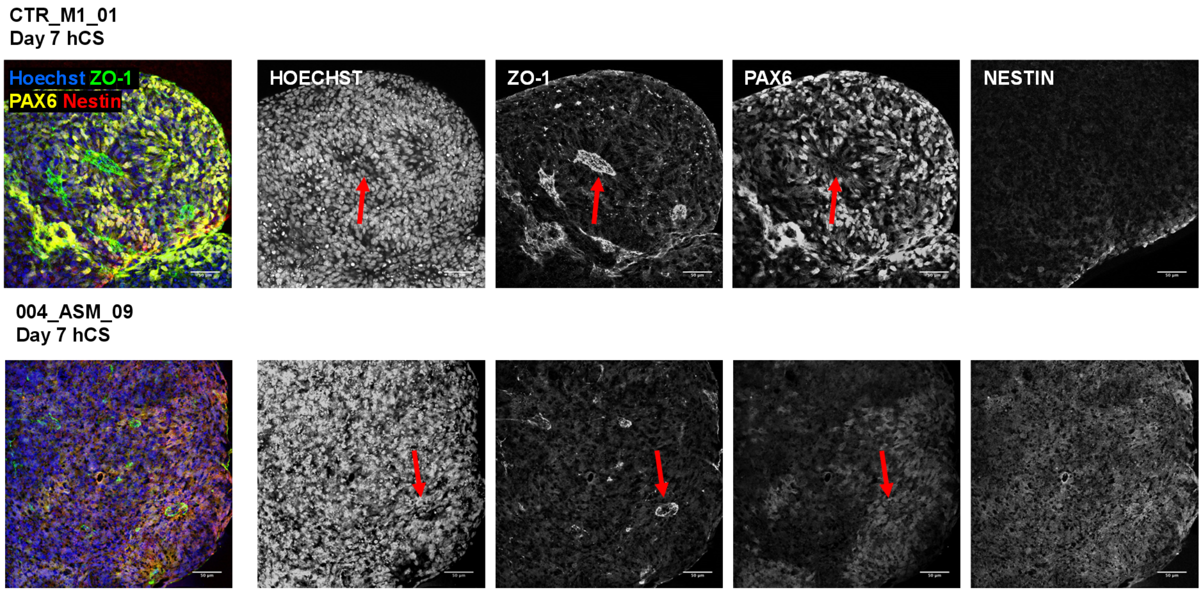

Supplemental Figure 3. Confocal images of day 17 hCS generated from typical (control) or autism hiPSCs. (A) Representative confocal images of sectioned (20 µm) day 17 control hCS. ZO-1 staining of apical membrane (red arrow) is surrounded by apico-basal polarised Nestin filaments and self-organised Pax6-positive cells. **(B)** Representative confocal image of sectioned day 17 autism hCS. ZO-1 staining of apical membrane (red arrow) is disrupted and nestin- and Pax6-positive cells do not display radial self-organised around the apical membrane as seen in control hCS.

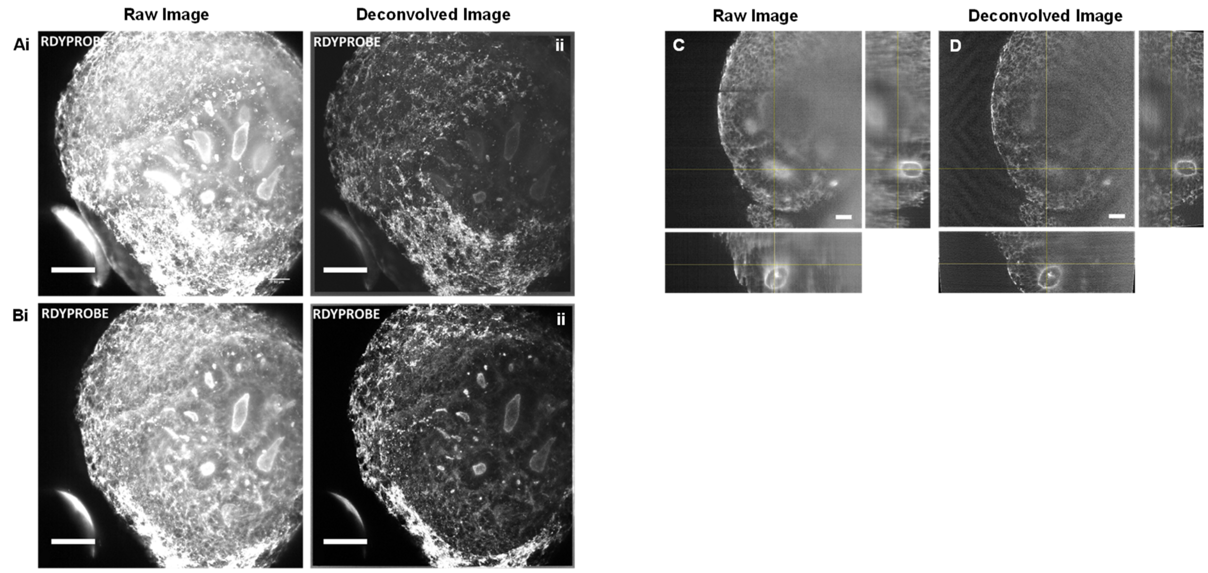

Supplemental Figure 4. Comparison of raw and deconvolved light sheet acquisitions of hCSs. (A) A raw **i)** and deconvolved **ii)** output max intensity Z stack, showing the full stack (500z, 200 µm) from acquisition including the outer edges of tissue. **(B)** A raw **i)** and deconvolved **ii)** section of the previous Z stack A) (60z-360z), removing the outer and deep edges of tissue permitting unobstructed visualisation of internal structures. Scale bars, 100 µm. Phallodin-488 Readyprobe (F-actin) on Day 30 hCSs. **(C)** An orthogonal view of a raw output Z stack image displaying cortical rosettes. **(D)** An orthogonal view of the same Z stack image as **(C)** but deconvolved. Scale bars = 50 µm.

A

B

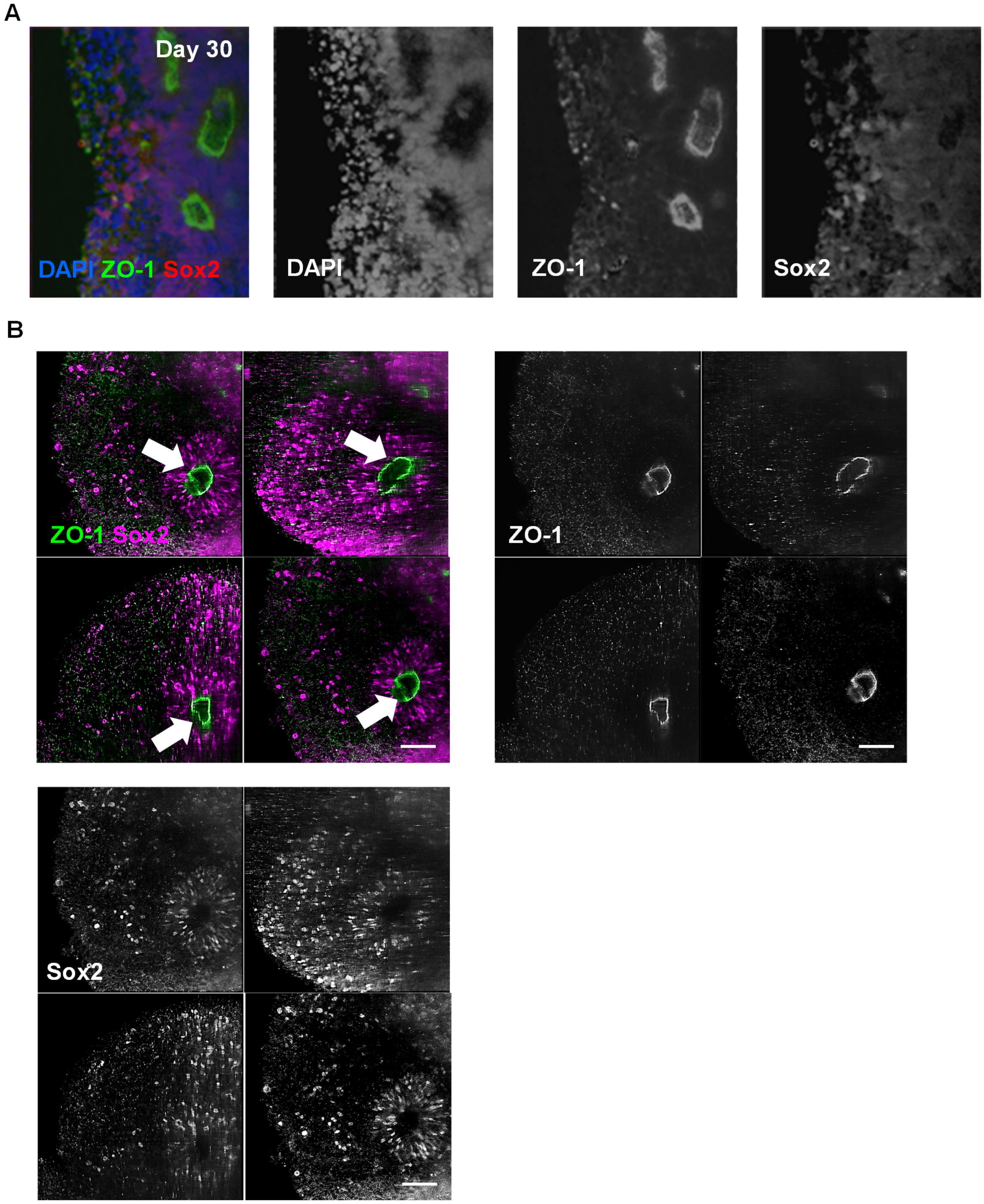

Supplemental Figure 5. ALSM imaging of cleared day 30 hCS. (A) Maximum intensity Z stack (2D) projections (composite and individual channels), showing the full stack of cleared day 30 hCS immunostained for ZO-1 (green), Sox2 (red) and DAPI (blue). **(B)** Maximum intensity Z stack (2D) projections of optical sections shown in main Figure 5 C. Images are composite for ZO-1 (green) and Sox2 (magenta)

channels, and subsequently of ZO-1 and Sox2 channels individually as grey scale images. Scale bars = 100 µm.

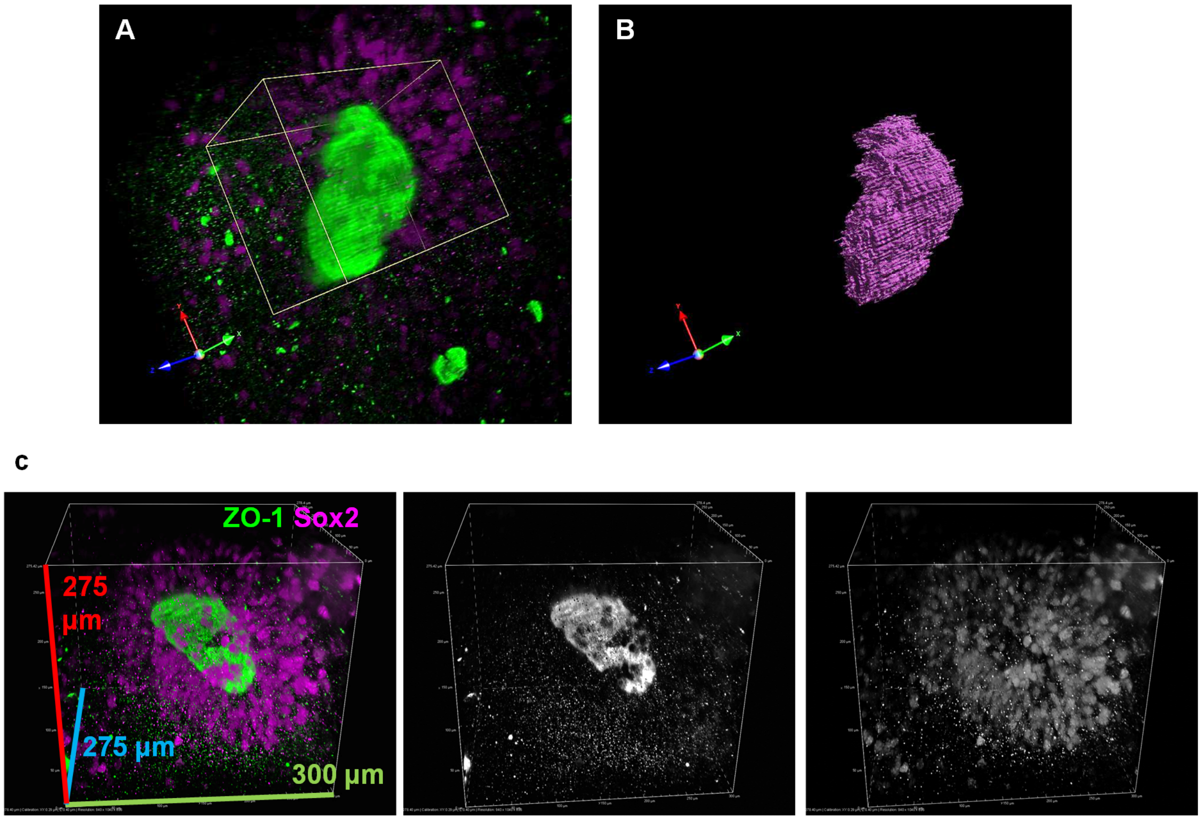

Supplemental Figure 6. ALSM imaging of neural rosette lumens in cleared day 30 hCS. (A) Alternative digital zoom of largest neural rosette lumen shown in main Figure 6E. Green channel indicates ZO-1-psotive apical membrane; magenta channel indicates Sox2-posiive NPCs surrounding central lumen. **(B)** Threshold image of ZO-1-positve lumen demonstrating morphology of structure in 3D. **(C)** Alternative representative 3D render of largest neural rosette lumen (shown in main figure 6G) demonstrating the radial organisation of Sox2-positve NPCs surrounding ZO-1-positive (green) rosette lumen.

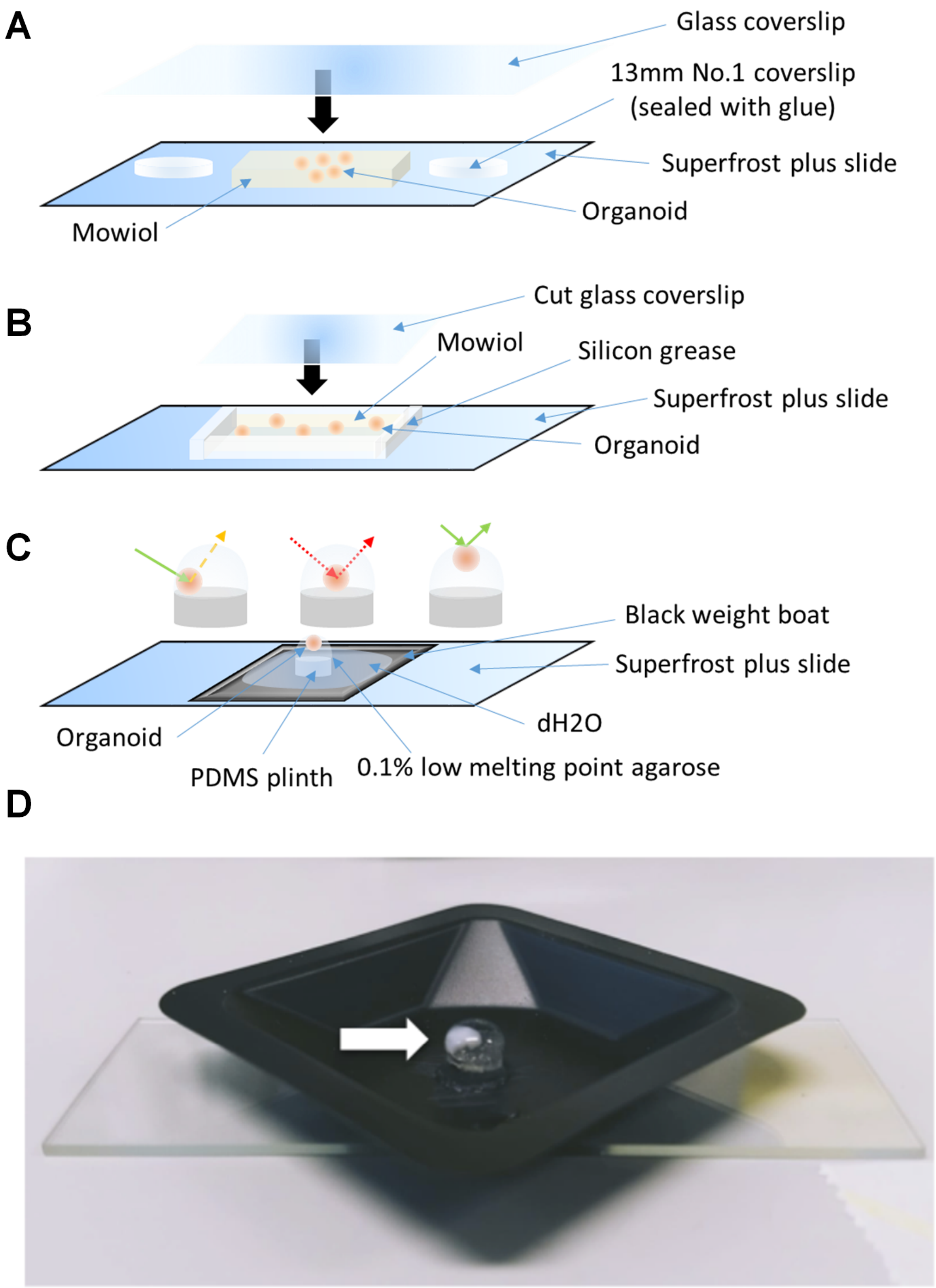

Supplemental Figure 7. Preparations of setups used for mounting intact 3D tissues. Figure displaying the mounting setups developed for imaging of

spheroids by confocal and ALSM. (A) The prototype setup used for initially imaging Day 7 hCS using a confocal microscope. Despite sealing the spheroids between the glass, Mowiol was free to exit the setup if not laid flat upon storage, in addition spheroids were larger than initially predicted (~300μm) thus may have been distorted as No.1 coverslips are only 100μm thick. **(B)** An improved design of **(A)** that uses a layer of silicon grease surrounding the spheroids to buffet incoming pressure. However, this method does not offer the same level of protection as hardened glass and ultimately Mowiol solution can leak if pressure is too intense. **(C)** A setup designed by M Squared Life for use with their ALSM. A continuous film of water must cover both the agarose and the objectives used for imaging to reduce visual aberrations. In addition, it is of utmost importance to place the sample tissue as close to the peak of the agarose hemisphere as possible, as to prevent the distance that laser focused light must travel before being refracted into the emitter, and to ensure the lowest amount of light scatter to reduce background signal. **(D)** A second iteration of the imaging setup for use with ALSM. This setup was also used for clearing of hCSs prior to immunostaining and subsequent imaging.

Supplemental Movie 1. Three-dimensional 'fly-through' of ALSM volumetric image of cleared day 30 hCS immunostained for NPC marker Sox2 (magenta) and apical membrane marker ZO-1 (green).

Supplemental Movie 2. Three-dimensional 'fly-through' digitally zoomed onto largest neural rosette lumen of cleared day 30 hCS. HCS were immunostained for NPC marker Sox2 (magenta) and apical membrane marker ZO-1 (green).

Supplemental Table 1: Primary antibodies used in study.

**Primary Antibody**	**Species**	**Supplier**	**Cat. No.**
Pax6	Rb	BioLegend	901301
Tuj1	Ms	BioLegend	801201
N-Cad	Ms	Sigma Aldrich	C3865
ZO-1	Rb	Thermo Fisher	40-2200
PKCλ	Ms	BD Bioscience	610207
DCX	Rb	Abcam	ab18723
MAP2	Chk	Abcam	ab92434
Nestin	Ms	Merck Millipore	MAB5326
Sox2	Rb	Merck Millipore	2003600

Supplemental Table 2: ScaleS solution components used in study.

**Ingredients**	**ScaleSQ(5)**	**ScaleS4(0)**
D-(–)-sorbitol (w/v)%	22.5	40
Glycerol (w/v)%	-	10
Urea (M)	9.1	4
Triton X-100 (w/v)%	5	-
Dimethylsulfoxide (v/v)%	-	25
pH	8.2	8.1
